# Telecardiology Application in Jordan: Its Impact on Diagnosis and Disease Management, Patients' Quality of Life, and Time- and Cost-Savings

**DOI:** 10.1155/2014/819837

**Published:** 2014-10-27

**Authors:** Yousef Saleh Khader, Mohamad Ismail Jarrah, Abde-Ellah M. Al-Shudifat, Amjad Shdaifat, Husham Aljanabi, Shadwan Ismeil Al-Fakeh, Elias Emil Turk, Khaled Ali Zayed, Hanadi A. Al Quran, Ziad Mohd Ellauzi, Mohammad Al Tahan

**Affiliations:** ^1^Department of Community Medicine, Public Health and Family Medicine, Faculty of Medicine, Jordan University of Science and Technology, P.O. Box 3030, Irbid 22110, Jordan; ^2^Jordan University of Science and Technology, Irbid, Jordan; ^3^The Hashemite University, Zarqa, Jordan; ^4^Ministry of Health, Amman, Jordan

## Abstract

*Objectives*. To assess the impact of live interactive telecardiology on diagnosis and disease management, patients' quality of life, and time- and cost-savings.* Methods*. All consecutive patients who attended or were referred to the teleclinics for suspected cardiac problems in two hospitals in remote areas of Jordan during the study period were included in the study. Patients were interviewed for relevant information and their quality of life was assessed during the first visit and 8 weeks after the last visit.* Results*. A total of 76 patients were included in this study. Final diagnosis and treatment plan were established as part of the telecardiology consultations in 71.1% and 77.3% of patients, respectively. Patients' travel was avoided for 38 (50.0%) who were managed locally. The majority of patients perceived that the visit to the telecardiology clinic results in less travel time (96.1%), less waiting time (98.1%), and lower cost (100.0%). Telecardiology consultations resulted in an improvement in the quality of life after two months of the first visit.* Conclusions*. Telecardiology care in remote areas of Jordan would improve the access to health care, help to reach proper diagnosis and establish the treatment plan, and improve the quality of life.

## 1. Introduction

Telemedicine is the use of information and communication technology to provide health care services for people in remote areas where the health care providers are not available [1]. Telemedicine has been shown to be feasible [2] and cost-effective [3, 4] and to provide organizational benefits and to improve patients' satisfaction [5].

Telecardiology, an application of telemedicine in cardiology, has been shown to improve the standard of cardiac care by providing diagnostic support for health professionals to safely manage their patients [6]. Studies have demonstrated that telecardiology improves the management of cardiac diseases in remote and rural populations in a cost-effective manner, reduces the number of unnecessary hospital referrals and admissions, results in avoidance of travel for patients in rural and remote areas, and improves health professionals' knowledge base [7–11]. Meta-analysis studies showed that telemedical monitoring of patients with chronic heart failure can improve overall survival by 17% to 47% during 6 to 12 months of follow-up [7, 8]. Moreover, some systematic reviews showed that telemedicine has a beneficial effect on health related quality of life among patients with heart failure [12–14].

In Jordan, many patients with cardiac diseases in the rural and remote locations receive cardiac care by general practitioners because of the scarcity of cardiologists in these areas. Other patients need to travel to receive these services in tertiary hospitals in Amman, the capital of Jordan. Because of geographic distance and costly travel, many patients find referral to a cardiologist very difficult. Thus, provision of telecardiology services in remote areas in Jordan could have its greatest impact. A telemedicine initiative was undertaken through the Jordan Healthcare Initiative (JHI) to facilitate access to quality healthcare services for patients and clinicians from two remote hospitals in northern and southern regions in Jordan to specialists at a tertiary hospital in Amman. This study was conducted to assess the impact of live interactive telecardiology consultations on diagnosis and disease management and assess if telecardiology was associated with improvement in patients' quality of life and time- and cost-savings.

## 2. Methods

### 2.1. Jordan Healthcare Initiative

Two teleclinics have been successfully implemented and are fully operational in two remote hospitals in north and south of Jordan. The first clinic was launched in 2011 at Mafraq Governmental Hospital (MGH) in the north of Jordan and the second was launched in 2012 at Queen Rania Hospital (QRH) in the south of Jordan. The two clinics connect patients from the north and south of Jordan with specialists at Prince Hamzah Hospital (PHH) in Amman.

### 2.2. Study Population and Design

A pretest-posttest one-group design was used to evaluate the process and outcomes of the teleconsultations that took place in the period between September 2013 and January 2014. This study focused on telecardiology and its impact. All consecutive patients who attended or referred to the teleclinics for suspected cardiac problems in both hospitals during the study period were included in the study. For each patient, the physician or the nurse in the remote hospitals filled the consultation request form and scheduled an appointment for the patient in the teleclinic within one week of the patient's first visit. In the day of the visit to the teleclinic and at the end of consultation session, the study team (two nurses in each hospital) interviewed patients using face-to-face interview and filled all study questionnaires. The quality of life questionnaires were filled again after 8 weeks of the last visit to the clinic using phone interview. All efforts were made to ensure that the phone interview is conducted according to prespecified protocol and guidelines set by the principle investigator to ensure high data quality and to minimize the losses to follow-up. The sample size that was needed to detect a medium effect size in the change of quality of life for patients with cardiologic conditions after receiving the teleconsultation at a power of 80% and level of significance of 0.05 was calculated as 53 subjects. The ethical approval was obtained from the institutional review board at Jordan University of Science and Technology.

### 2.3. Data Collection

Different aspects were assessed in the evaluation process. All questionnaires and forms used in this study were identified based on the review of the relevant studies. English questionnaires were translated into Arabic using a forward-backward translation method and were subsequently adapted to the Jordanian culture. The questionnaires were pilot tested in 10 patients and the necessary changes were made. All nurses were trained on data collection methods including phone interview and face-to-face interview, on ethical aspects of research conduct, and on all study procedures.

### 2.4. Consultation Request Form

This form was filled for each patient during the initial visit by the physician or the trained nurse in the remote hospitals and a copy was sent to the specialists in the main hospital by fax or email. The form included information about the patient's demographics, chief complaint, disease or condition category, medical history, current medications, and provisional diagnosis and treatment plan. All necessary and available documents such as lab results that may help to reach the diagnosis or the proper treatment and follow-up of the patient were sent to the specialist.

### 2.5. The Record Form

This form was filled by the physicians or trained nurses in the remote hospitals at the end of the consultation. The record form included information about the final diagnosis and proper treatment plan as agreed upon by the physician and the specialist. One question was used to assess the physician perception about whether the communication with the specialist helped the physician to reach better diagnosis and/or treatment plan. A change in diagnosis was defined as whether the consultant cardiologist's diagnosis was different from that of the referring physician's diagnosis at the completion of the initial telecardiology consultation. A change in disease management was defined as whether the cardiologist recommended treatment plan that was different from what was recommended by the referring physician.

### 2.6. Patients' Perception and Satisfaction Questionnaire

A structured questionnaire was filled using face-to-face interview at the end of the teleconsultation session to assess the patients' perception and satisfaction with the telemedicine application and determine its effect on the quality of care and health outcomes. The questionnaire consisting of 15 items was divided into different sections to give a complete picture on different domains including medical improvement, time- and cost-savings, the telemedicine preparation, proper case management, and diagnosis and treatment. Likert scale of five responses was used to rate the individual items in each domain with higher scores indicating better satisfaction. The total satisfaction score was calculated by summing the responses of all individual items in the questionnaire and transformed to a score on 0–100 scale.

### 2.7. Quality of Life Questionnaires

One generic quality of life questionnaire (Arabic version of the short form (SF-8) questionnaire) [14] and selected questions from the Minnesota Living with Heart Failure Questionnaire (LIhFE) [15] for patients with heart disease were used to assess the quality of life. The SF-8 was constructed to replace the SF-36 and SF-12 in population health surveys in the U.S. and internationally. Accordingly, it has been translated and linguistically validated for use in more than 30 countries and languages including Arabic language using IQOLA Project methods. The LIhFE has been translated into 33 languages including Arabic language (even though some of the translations may not have undergone a full linguistic validation methodology). For the LIhFE, the patient graded each question from 0 to 5. The patients' judgment was based on how much his/her heart condition influenced the specific activity over the last week. The questionnaires were filled in the same day of the teleconsultation using face-to-face interview method and the same questionnaires were filled 8 weeks later using phone interview method to assess the changes in the quality of life as a result of teleconsultation. SF-8 consists of eight items, each representing one health profile dimension: general health perception, physical functioning, role functioning-physical, bodily pain, vitality, social functioning, mental health, and role functioning-emotional. Each item of the SF-8 was assessed using a 5- or 6-point Likert scale. Each item of SF-8 and LIhFE was then scored on a 0 to 100 range so that the lowest and highest possible scores are 0 and 100, respectively, with a high score of SF-8 and low score of LIhFE defining a more favorable health state. The SF-8 scale score and LIhFE score represent the average for all items in the scale that the respondent answered.

### 2.8. Statistical Analysis

Data were described and analyzed using the IBM SPSS Statistics (version 20). Data were described using means, standard deviations, or percentages wherever appropriate. Improvement in quality of life after telemedicine application compared to the baseline was analyzed using paired *t*-test. A *P* value of less than 0.05 was considered statistically significant.

## 3. Results

### 3.1. Patients' Characteristics

A total of 76 patients (53.9% males and 46.1% females) attended the telecardiology clinics in the two remote hospitals between September 2013 and January 2014. The demographic and relevant characteristics of these patients are shown in Table 1. The age of patients ranged from 18 to 93 years with a mean (SD) of 49.0 (13.4) year. Less than one-third of patients (30.3%) were younger than 45 years. The majority of telecardiology consultations were done in MGH and the majority of patients (81.6%) were referred from outside clinic/center. Approximately one-half of patients (51.3%) had hypertension and one-third (33.3%) had diabetes mellitus. About 71.1% of patients complained of chest pain and 53.3% complained of shortness of breath.

### 3.2. The Impact of Telecardiology Consultations on Diagnosis and Treatment Plan

Establishing or helping in diagnosis or treatment plan was the main reason for teleconsultations in all patients. As perceived by the referring providers, final diagnosis was established as part of the telecardiology consultations in 71.1% of patients and changed from that of the referring provider in 17.1% of patients. The diagnosis remained the same as that of the referring provider in 11.8% of patients (Table 2). The treatment plan was established for 77.3% of patients as part of the teleconsultations and changed from that of the referring provider for 16.0% patients. The referring providers perceived that the communication with the specialist helped them to reach the diagnosis and the treatment plan in all consultations.

According to the final diagnosis, about half of patients (52.6%) had ischemic heart disease, 35.5% had noncardiac problems, 5.3% had heart failure, 5.3% had cardiomyopathy, and 2.6% had valvular heart disease. As a result of teleconsultations, 24 (31.6%) patients were treated locally in the same visit, 14 (18.4%) were scheduled for a follow-up by the specialist via teleclinic and treated, 8 (10.5%) were referred for outpatient diagnostics outside the region, 6 (3.9%) were referred to specialized consultants outside the region for evaluation and treatment, and 24 (31.6%) were referred for procedures and/or hospitalization.

### 3.3. Patients' Perception of the Time- and Cost-Savings and Their Satisfaction

Overall, patients' travel was avoided for 38 (50.0%) who were managed locally. While none of the patients perceived that it is easy to access the specialist clinic in Amman, 98.7% of the patients stated that it was easy to access to the telecardiology clinic. The majority of patients perceived that the visit to the telecardiology clinic results in less travel time (96.1%), less waiting time (98.1%), and lower cost (100.0%) when compared to visiting the specialist clinic in the referral hospital (Table 3). When patients were asked about what they would do if the telecardiology was not available, 88.2% reported that they would travel to see the specialist, 10.5% will see a noncardiologist in the same hospital, and one patient reported that he would have done nothing about it. The mean (SD) waiting time in the teleclinic was 43.2 (24.7) minutes and the mean consultation time was 19.5 (12.8) minutes. The total satisfaction score of patients with the services received ranged from 65.4 to 100 with a mean (SD) of 94.6 (7.6) indicating a high level of satisfaction.

### 3.4. The Impact of Telecardiology Consultations on Quality of Life

A total of 50 patients had completed SF-8 and LIhFE measurements at the baseline and follow-up.  Table 4 shows the changes in the quality of life after two months of telecardiology consultations. After two months of telecardiology consultations, the mean SF-8 score increased significantly from 40.7 to 62.4 (*P* < 0.005) with a mean change from the baseline of 21.7 (95% confidence interval: 15.3, 28.0). This implies that the telecardiology consultations resulted in an improvement in the quality of life and resulted in a more favorable health state. All SF-8 domains had improved significantly after two months of telecardiology consultations except mental health domain. The highest improvements were seen in social functioning and role-emotional domains of SF-8. Moreover, the baseline mean LIhFE score decreased significantly from 47.8 to 34.9 after two months of telecardiology consultations indicating that there had been a meaningful improvement in patient's quality of life since the previous measurement of LIhFE scores at the baseline.

## 4. Discussion

Various medical specialties use telemedicine to provide care to remote populations, thereby minimizing travel and reducing costs. Telemedicine has a promising application in the diagnosis and management of cardiac diseases in remote and rural areas [16]. However, there is still a debate on the benefits and effectiveness of telemedicine. To be adopted into everyday practice, telemedicine should show improved access to healthcare, improved quality of life, and cost-savings. Therefore, these issues were considered in the evaluation of telemedicine application in Jordan.

Telemedicine enables the remote exchange of data between patients and healthcare professionals to facilitate diagnosis, monitoring, and management of conditions [17, 18]. Because of the shortage of cardiologists in remote and rural areas in Jordan, the majority of patients with cardiac problems in these areas need to travel about 100–300 Kilometers to the tertiary hospitals in Amman to receive the care. This study showed that such costly travels might be avoided for some patients as almost one-third (35.5%) of patients who attended the teleclinic had noncardiac problems and 50.0% were treated locally without a need for traveling. Besides, this study showed that the telecardiologists helped to establish the final diagnosis in 71.1% of patients.

Another option for patients in the absence of teleclinic is to receive the care by noncardiologists. This option has implications on the diagnosis and treatment. In about 17.1% of patients, the initial diagnosis made by the referring providers had been changed. Telecardiology helped to establish the treatment plan for 77.3% of patients and it has been changed from that of the referring provider for 16.0% patients. Therefore providing telecardiology care in remote areas of Jordan would improve the access to health care and help to reach proper diagnosis and establish the treatment plan.

Avoidance of travel, by patients, their relatives, and health care professionals, is a major benefit of telecardiology. The majority of patients in this study perceived that the visit to the telecardiology clinic resulted in less travel time, reduced waiting time, and lower cost when compared to visiting the specialist clinic in Amman. Previous studies showed that telemedicine in remote areas may save costs by reductions in travel time [19, 20]. Klinkman et al. showed that 75% of all analysed chronic chest pain referrals from GPs to cardiologists were due to noncardiac causes, being either musculoskeletal or nonspecific in origin [21], and thus the travel can be avoided for a high percentage of patients.

Our study showed that telecardiology consultations resulted in a meaningful improvement in patient's quality of life since the previous measurement of SF-8 and LIhFE scores. All domains of SF-8 improved after two months of treatment. Another pre/poststudy of telemedicine found significant improvements in role-physical, bodily pain, and social functioning domains of quality of life [22]. On the other hand, some other studies that actually measured health related quality of life found no difference between telephone support and usual care [23]. Systematic reviews that have examined the effect of telemedicine on health related quality of life in heart failure showed that telemedicine is beneficial [13, 24, 25].

This study was not intended to compare telecardiology to in-person cardiology care because telecardiology may still be superior to cardiologic care provided by nonspecialists. In conclusion, telecardiology care in remote areas of Jordan would improve the access to health care, help to reach proper diagnosis and establish the treatment plan, and improve the quality of life. A study with a comparison group of patients who used the in-person cardiology care would provide stronger evidence on the effectiveness of telecardiology care.

## Figures and Tables

**Table 1:** The characteristics of patients who attended the telecardiology clinics in Mafraq Governmental Hospital (MGH) and Queen Rania Hospital (QRH) between September 2013 and January 2014.

Variable	*n*	%
Age (year)		
<45	23	30.3
45–54	32	42.1
≥55	21	27.6
Sex		
Male	41	53.9
Female	35	46.1
Hospital		
MGH	68	89.5
QRH	8	10.5
Source of patients		
Outpatient clinic	62	81.6
Inpatient	9	11.8
Outside clinic/center	5	6.6
Smoking	9	12.0
Family history of cardiovascular diseases	12	15.8
Medical history		
Myocardial infarction	5	6.6
Chronic obstructive pulmonary disease	2	2.6
Hypertension	39	51.3
Diabetes mellitus	25	33.3
Symptoms		
Chest pain	54	71.1
Shortness of breath	40	53.3
Palpation	10	13.2
Weakness	5	6.6
Arrhythmia	4	5.3

**Table 2:** The impact of telecardiology consultations on changes in diagnosis and treatment plan according to the perception of the referring providers.

The impact	*n*	%
Diagnosis		
Established as part of the telecardiology consultation	54	71.1
Remained the same as the initial diagnosis	9	11.8
Changed as a result of the telecardiology consultation	13	17.1
Treatment plan		
Established as part of the telecardiology consultation	58	77.3
Remained the same as the initial plan	5	6.7
Changed as a result of the telecardiology consultation	12	16.0

**Table 3:** Patients' perception of the visit to the telecardiology clinic in terms of travel time, waiting time, and cost as compared to visiting the specialist in the referral hospital.

Perception	*n*	%
Travel time		
Same travel time	0	0
More travel required	3	3.9
Less travel required	73	96.1
Waiting time in the clinic		
Same waiting time	1	1.3
Increased waiting time	0	0
Reduced waiting time	75	98.7
Cost		
Same cost	0	0
Greater cost	0	0
Lower cost	76	100.0

**Table 4:** The changes in the quality of life among 50 adult patients after two months of telecardiology consultations as measured by SF-8 and its domains and LIhFE^*^.

Measures of quality of life	Baseline (before) Mean (SD)	After 8 weeks (after) Mean (SD)	Changes in score (before-after)∗∗ Mean (95% confidence interval)	*P* value
Total SF-8 score	40.7 (18.0)	62.4 (19.6)	21.7 (15.3, 28.0)	0.000
General health	37.2 (21.4)	68.8 (22.2)	31.6 (22.5, 40.7)	0.000
Physical functioning	36.0 (25.3)	53.5 (35.7)	17.5 (6.5, 28.5)	0.003
Bodily pain	36.7 (24.0)	52.0 (36.0)	15.3 (4.1, 26.5)	0.009
Role-physical	41.2 (20.4)	60.8 (36.1)	19.6 (8.3, 30.9)	0.001
Vitality	43.1 (19.3)	60.1 (30.2)	17.0 (6.3, 27.7)	0.003
Social functioning	44.4 (23.5)	85.2 (25.5)	40.8 (32.7, 48.9)	0.000
Mental health	42.0 (20.5)	37.5 (36.9)	−4.5 (−7.2, 16.2)	0.444
Role-emotional	43.9 (20.7)	80.6 (30.3)	36.7 (27.6, 45.9)	0.000
Total LIhFE scores	47.8 (22.1)	34.9 (20.7)	−12.8 (−6.4, −19.3)	0.000

^*^SF-8: Medical Outcomes Survey Short Form-8 questionnaire; LIhFE: selected questions from Minnesota Living with Heart Failure Questionnaire.

∗∗Positive sign for the change in SF-8 and its domains score and negative sign for changes in LIhFE score indicate improvement in quality of life and health state.
